# Effects of resistance exercises on rotator cuff muscle mechanical characteristics in shoulders with and without rotator cuff tears

**DOI:** 10.1371/journal.pone.0347233

**Published:** 2026-05-27

**Authors:** Andrew J. Nasr, Henry Wang, Jijia Wang, Michael Khazzam, Nitin B. Jain, Yen-Sheng Lin

**Affiliations:** 1 School of Physical Therapy, Texas Woman’s University, Dallas, Texas, United States of America; 2 Department of Neuroscience, Brown University Providence, Rhode Island, United States of America; 3 Department of Applied Clinical Research, UT Southwestern Medical Center, Dallas, Texas, United States of America; 4 Baylor Scott & White, Orthopaedic Associates of Dallas, Complex Shoulder Institute, Grapevine, Texas, United States of America; 5 Department of Physical Medicine and Rehabilitation, University of Michigan, Ann Arbor, Michigan, United States of America; 6 Department of Orthopaedic Surgery, UT Southwestern Medical Center, Dallas, Texas, United States of America; 7 Department of Physical Medicine and Rehabilitation, UT Southwestern Medical Center, Dallas, Texas, United States of America; 8 Graduate Institute of Medical Sciences, National Defense Medical University, Taipei, Taiwan; Carol Davila University of Medicine and Pharmacy: Universitatea de Medicina si Farmacie Carol Davila din Bucuresti, ROMANIA

## Abstract

**Background:**

Rotator cuff tears are the primary cause of shoulder pain, often managed with resistive exercise. Understanding how rotator cuff muscles respond to low-intensity rehabilitation-based exercise is critical for optimizing rehabilitation strategies, yet these responses remain poorly characterized.

**Methods:**

This study investigated changes in shear wave speed, cross-sectional area, and strength of the supraspinatus and infraspinatus muscles following a 30-repetition isometric contraction protocol at 20% of isometric volitional contraction in shoulders with and without rotator cuff tears. Three-way mixed ANOVAs with repeated measures were performed to evaluate the main effects of tear status, baseline vs post-exercise, passive and active conditions on supraspinatus and infraspinatus as well as the interaction between main effect and cross-sectional area and shear wave speed.

**Results:**

Twenty participants were assessed using ultrasound imaging pre- and post-exercise. The findings revealed that participants with rotator cuff tears experienced a significant reduction in passive supraspinatus shear wave speed (*p* = 0.0021), indicative of increased muscle compliance, while asymptomatic participants exhibited no significant change. Cross-sectional area increased significantly post-exercise in both groups under active conditions (asymptomatic group: *p* = 0.0046 and symptomatic group: *p* = 0.0020), though no change was observed in rotator cuff tear participants during passive testing (*p* = 0.8900). Strength assessments showed marked declines in both groups following exercise (*p*-values <0.005), reflecting peripheral weakness.

**Conclusion:**

These findings suggest that rotator cuff tears are associated with maladaptive reductions in supraspinatus muscle stiffness during acute exercise, potentially impairing functional capacity and necessitating longer recovery periods. In contrast, asymptomatic participants maintain supraspinatus stiffness despite weakness, underscoring differential adaptation to low-intensity exercise.

## Introduction

Shoulder pain is a common musculoskeletal complaint globally, exhibiting a point prevalence of 26%. [1,2] Around 66.7% of adults are likely to experience shoulder pain at some point in their lives, with rotator cuff tears (RCTs) identified as the primary etiology. [3] An initial trial of non-operative treatment is often recommended, with resisted exercises aimed at improving glenohumeral motion and active scapular retraction, followed by exercises to improve scapular and glenohumeral muscle function, as the preferred intervention based on clinical practice guidelines. [4–6] Resistive exercise has been demonstrated to significantly improve patient outcomes however, the incidence of nonresponse rates remain concerning. [7] This variability in individual response underscores the necessity for a nuanced understanding of the underlying mechanisms contributing to such variability. A recent scoping review examining exercise programs for managing rotator cuff-related pain highlighted considerable variability in the frequency, intensity, time, and type of exercise utilized in randomized controlled trials, emphasizing inconsistency of physical therapy intervention. [8] Rotator cuff disease is multifactorial with recent theoretical models suggesting distinct mechanistic domains as it relates to a patient’s response to resisted exercises. [9] There remains clinical uncertainty in how the rotator cuff muscles respond to submaximal exercise in healthy populations and in patients with RCTs.

In healthy populations, muscle cross-sectional area (CSA) increases [10,11], and strength decreases [12,13] following an acute bout of high-intensity exercise (e.g., pitching). However, the acute effects of submaximal exercise on muscle strength and volume in compromised populations are less understood. Much of the existing literature focuses on biomechanical alterations secondary to fatigue, utilizing electromyography studies. Surface electromyography effectively captures muscle activity, however, it is constrained by its inability to isolate specific muscles, often recording signals from adjacent musculature due to intermuscular crosstalk. [14] Shear wave elastography (SWE), on the other hand, is an ultrasound-based technique for investigating the mechanical properties of skeletal muscle. [15] SWE is capable of avoiding intermuscular crosstalk and has a high coefficient of determination (R^2^ = 0.94–0.96) with electromyography. [16,17] Briefly, SWE provides a tissue stiffness measure based on the speed at which the shear waves propagate across a medium, defined as shear wave speed (SWS, in m/s) when induced by an acoustic radiation pulse force. [18] SWE has been well studied as a tool to assess muscle fatigue or weakness in humans, however, much of this work has been on young healthy populations. [19–21] Ultrasound imaging is a reliable modality to measure rotator cuff muscle CSA. [22–25] However, the effects of the low intensity exercises on both CSA and SWE measures of rotator cuff muscle stiffness in the setting of RCTs are unknown. Improving our understanding of how the rotator cuff muscles respond to low-intensity exercises commonly prescribed in rehabilitation may help identify phenotypes that are more or less likely to benefit from traditional progressive resistive exercise. The purpose of this study was to quantitatively assess changes in rotator cuff muscle architecture following a low-intensity exercise protocol in participants with and without RCTs.

This study aimed to investigate the time course of shear wave elastography-derived SWS, muscle cross-sectional area, and strength of the supraspinatus and infraspinatus muscles following a 30-isometric contraction exercise protocol at 20% of the calculated maximal voluntary isometric contraction. We hypothesized that SWS and strength would decrease while CSA would be unchanged following low-intensity exercise in subjects with MRI-confirmed RCTs, indicating greater muscle compliance (reduced muscle stiffness) and impaired muscle function. Our secondary hypothesis was that the asymptomatic population would have no change in SWS, CSA, or strength following low-intensity exercise.

## Methods

### Participants

All participants signed informed consent of the experimental procedures and gave written consent before any testing was conducted. No participant was included in both the rotator cuff tear and asymptomatic group. A sample size of 18 was deemed sufficient to determine statistical power. This power calculation was completed using G*Power 3.1.9.6. A moderate effect size of 0.30 was selected based on previous data by Siracusa et al. where they quantified muscular fatigue of the quadriceps muscle utilizing SWE imaging by enrolling 15 subjects to detect an effect size of 0.40 to achieve 95% power at a significance level of 0.05. [19] Participant recruitment for this study began on 1 April 2024 and the final participant was enrolled on 8 August 2024. Each participant completed an eligibility session via email or phone call. Due to the nature of the study and the use of ultrasound imaging, blinding of participants and the sonographer was not feasible. In addition, as this was an observational rather than an interventional study, group randomization was not performed. This study was approved by the University Institutional Review Board and all experiments were conducted in accordance with the Helsinki Declaration. [26] Inclusion criteria for all case participants: (1) over the age of 40; (2) MRI or Ultrasound evidence of a full-thickness tear of the supraspinatus. Inclusion criteria for all control participants: (1) over the age of 40; (2) No history of shoulder trauma or episode of shoulder pain lasting greater than 72 hours in the preceding two years. Exclusion criteria for both groups included no history of shoulder surgery or neuromuscular condition affecting shoulder muscle health or function. Control participants did not undergo advanced imaging to confirm the absence of a RCT and therefore we defined this group as asymptomatic participants. At the time of data collection, no participant was receiving medication that could interfere with neuromuscular responses or had any neuromuscular disorders. Participants were asked to refrain from physical activity for at least 24-hours prior to data collection.

### Exercise protocol

**Fig 1:**
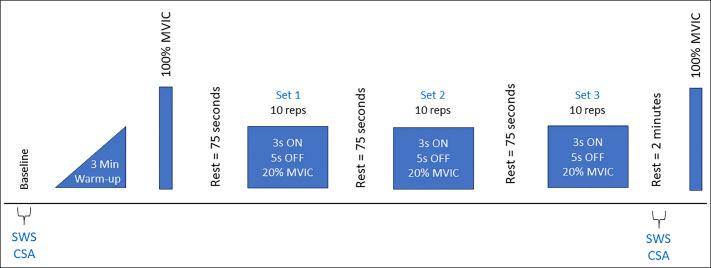
Exercise and Imaging Protocols. MVIC-maximal volitional isometric contraction; SWS-shear wave speed; CSA-cross-sectional area; Min-minute.

**Fig 2:**
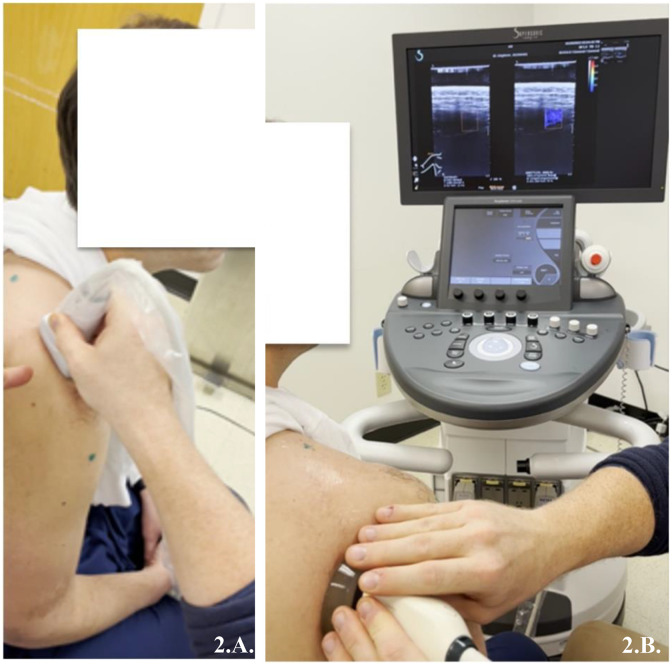
A. Testing position for the supraspinatus under passive conditions with the arm securely passive in the participant’s lap. B. Testing position for the infraspinatus under passive conditions with the arm passive in the participant’s lap.

**Fig 3:**
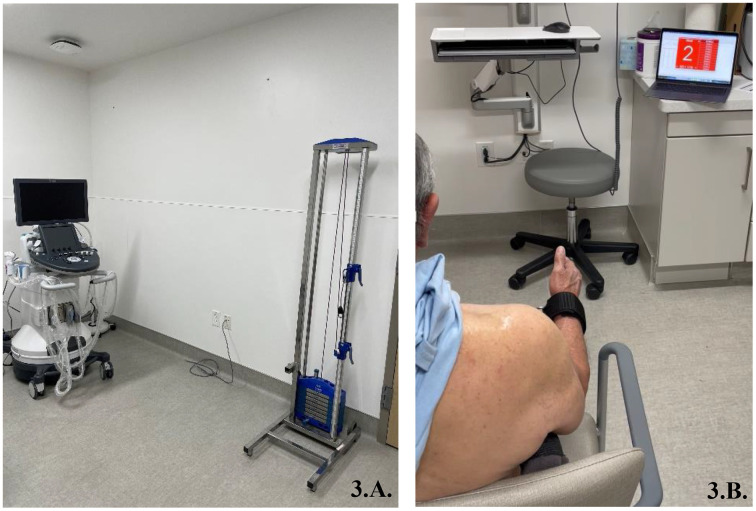
A. Ultrasound shear wave elastography and pulley weight system setup. B. Patient performing the external rotation isometric exercise protocol.

After a 5-minute warm-up at submaximal intensity, participants performed an intermittent voluntary exercise protocol consisting of 3 x 10 repetitions of alternating isometric 3-second contractions at 20% maximal volitional isometric contraction of the external rotation and a 5-second passive recovery between repetitions and 75-seconds between each set (Fig 1). Following each exercise, participants were asked to rate their perceived level of exertion from 1–10, a subjective measure of the amount of effort experienced during physical activity. [27] All participants were marked at a 1 at baseline prior to exercise (“hardly any exertion, but more than sleeping”). The number of contractions and percent of peak strength were chosen based on pilot testing and clinical experience to generate a significant level of voluntary strength loss without causing pain and discomfort in the RCT population. [28,29] The experimental exercise intensity closely resembles that experienced by patients with rotator cuff disease undergoing formal physical therapy for concentric external rotation strengthening exercises. A linear transducer was positioned accompanied by an external marker, outside of the region of interest and within the field of view, guiding probe placement over the supraspinatus (Fig 2A) and infraspinatus (Fig 2B) before and after the exercise. This approach facilitated the rapid identification of pertinent anatomy, thereby improving reliability prior to the acquisition of SWS measurements. Participants had visual and auditory cues on when to contract and when to relax. Fig 3 illustrates the experimental setup, showing a participant performing the exercise under the guidance of the prescribed protocol.

### Experimental setup and outcome measures

#### Muscle strength.

Muscle strength was assessed under isometric conditions with a MicroFET 3 hand-held dynamometer (Hoggan Scientific, LLC. Salt Lake City, Utah). To measure peak strength of the supraspinatus muscle, the device was fixed securely proximal to the wrist joint with a non-compliant strap maintaining the shoulder in 90^o^ of elevation in the scapular plane as described previously. [30] This position of shoulder external rotation at 90^o^ of shoulder elevation resulted in maximal neural activation of the supraspinatus. [30] The infraspinatus was tested with the participant seated in a chair with a towel roll under the axilla and the shoulder at 0^o^ of abduction and neutral rotation as previously described. [31] Peak isometric strength was calculated from the average of two trials. None of the participants reported pain during any of the trials, however one rotator cuff tear participant was unable to generate measurable supraspinatus force against the dynamometer and therefore missing data was not including in the final analysis. Following the completion of the exercise protocol, maximal voluntary contraction was reassessed after a minimum recovery period of 2 minutes to allow for metabolic recovery. [32] The testing parameters and instructions used were consistent with those established during baseline testing.

#### Cross-sectional area.

**Fig 4:**
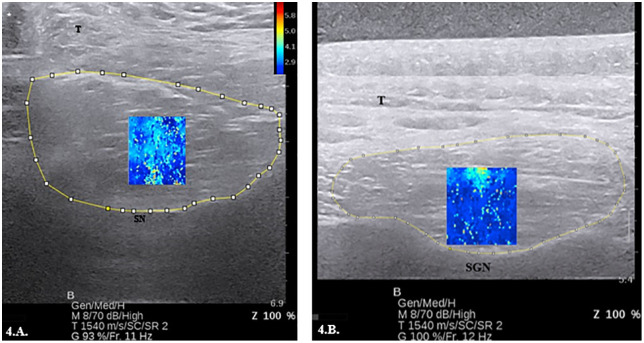
A. Shear wave elastography view of the supraspinatus muscle with selected region of interest and resulting color map. Shear wave speed range was set between 0.0 and 6.5 m/s. B. Shear wave elastography view of the infraspinatus muscle where shear wave speed range was set between 0.0 and 6.5 m/s. A higher speed corresponds to greater tissue stiffness (red) while a lower speed represents lower tissue stiffness (blue). T-trapezius muscle; SN-scapular notch; SGN-spinoglenoid notch; *-location of external marker.

Muscle cross-sectional area was measured using an Aixplorer Ultimate (SuperSonic Imagine, Aix-en-Provence, France) ultrasound unit with a SuperLinear™ transducer array (5−16 MHz, SL18−5 transducer, Supersonic Imagine Hologic, U.S.A). Images were captured in standard brightness mode on the default musculoskeletal setting. Participants were placed in a chair and instructed to sit with an upright posture with shoulders in a neutral position and hands placed comfortably in their lap as previously described by Yi et al. [23] All ultrasound images were performed by a licensed orthopaedic physical therapist (A.J.N.) with over 12 years of clinical experience and 3 years of training in shoulder sonography research. CSA measurements were taken under two testing conditions, active and passive. Active conditions for the supraspinatus involved muscle activation of the supraspinatus against gravity. Active conditions for the infraspinatus involved concentric external rotation against a non-elastic strap using a hand-held dynamometer to maintain a strength equal to 20% of the maximal volitional contraction measured during peak strength testing. Passive testing conditions required the participant to sit fully supported in a chair with upright posture and testing arm supported in their lap. For supraspinatus CSA measurements, the scapular notch was identified with the probe aligned parallel to the muscle fibers and then rotated along the short axis for image acquisition with the scapular notch centered on the monitor (Fig 4A). [33] An external marker was placed on the participants skin outside the region of interest to improve reproducibility of probe placement within the imaging session. For CSA measurements of the infraspinatus, the spinoglenoid notch was identified with the probe aligned parallel to the muscle fibers and then rotated along the short axis for image acquisition with the spinoglenoid notch centered on the monitor (Fig 4B). [33] To evaluate muscle CSA, DICOM files obtained from imaging were transferred to a dedicated workstation and processed using ImageJ software (National Institutes of Health, Bethesda, MD, USA). A minimum of 5 images for each participant were processed and the average was recorded. To convert the computed CSA from ImageJ’s default unit of pixels^2^ to cm^2^, a custom MATLAB function was utilized, standardizing measurements for analysis.

#### Shear wave elastography.

Participants remained in the same position for SWE imaging, with relevant anatomy identified in standard brightness mode before switching to SWE-mode using the default musculoskeletal setting. Images were captured under active and passive conditions for both muscles as previously described. For the supraspinatus region of interest determination, the scapular notch was again identified for image acquisition. For the infraspinatus, the spinoglenoid notch was again identified and centered on the screen for image acquisition. Care was taken to minimize compression of the probe against the skin to not influence the soft tissue. For each subject, five images were acquired for the supraspinatus and infraspinatus with at least 5 seconds between trials. Images were captured for each muscle at baseline and following each exercise set. Each image trial resulted in a brightness mode image and a corresponding SWE image where an internal proprietary software calculated shear wave speed (SWS; measuring range 0–10 m/s) at each pixel within a pre-selected region of interest, approximately 1.0 x 1.0 cm. The mean and standard deviation of shear wave speed within the region of interest were calculated using a custom MATLAB code (MathWorks Inc., Natick, MA, USA) and then averaged across the five trials.

### Statistical analysis

For continuous measurements, normality was assessed using the Shapiro-Wilk test, and normally distributed data were presented as the mean ± standard deviation. Demographic and clinical variables were compared between groups using either the independent t-test or the Mann-Whitney U test, as appropriate. Three-way mixed ANOVAs with repeated measures were conducted to examine the effects of rotator cuff status (symptomatic vs. asymptomatic), exercise (baseline vs. post-exercise), and SWE condition (active vs. passive) on SWS and CSA for each supraspinatus and infraspinatus muscle. Post-hoc analysis for main effects and interactions between CSA and SWS were conducted using Tukey’s HSD. Changes in muscle strength were evaluated using a paired t-test or Wilcoxon signed-rank, as appropriate. Intra-operator reliabilities for SWS and CSA measurements were assessed using an intraclass correlation coefficient based on a two-way mixed-effects model with single measurements and consistency agreement (ICC_**(2,3)**_). Reliability analyses were performed across repeated trials obtained within a single testing session for each muscle and testing condition (active and passive supraspinatus and passive infraspinatus). Measurement precision was further quantified by calculating the standard error of measurement (SEM), the minimum detectable difference (MDD), and the minimum detectable difference expressed as a percentage (MDD%) for the supraspinatus and infraspinatus muscles. The SEM, calculated as SEM = √(1 − ICC) × σ, where σ denotes the standard deviation of the measurements, reflects the expected variability of an observed score around the true value upon repeated assessment. The MDD was calculated as MDD = 1.96 × SEM × √2 and represents the smallest change that exceeds measurement error and can therefore be interpreted as a real difference. The significance level was set as 0.05. All data analyses were performed using SAS 9.4 (SAS Institute Inc., Cary, NC).

## Results

A total of 20 volunteers (32 shoulders) with and without MRI-confirmed RCTs completed the study protocol. Participant characteristics for the asymptomatic group (47%) were as follows: age: 67.67 ± 7.67 years, BMI: 26.89 ± 5.57 kg*m^-2^, Female: 7 (47%) and the symptomatic group age: 70.47 ± 6.18, BMI: 30.61 ± 6.60, Female: 11 (65%). All participants were right-hand dominant, except for one individual in the asymptomatic group who was left-hand dominant and one individual in the rotator cuff group who reported ambidexterity. The symptomatic group had an average time since rotator cuff tear diagnosis of 39.27 ± 25.67 months. Of the 17 rotator cuff tears, 8 (47%) were massive three-tendon tears and 7 (41%) involved two tendons. The supraspinatus tendon was torn in all the affected shoulders, with only 2 participants (12%) presenting with an isolated supraspinatus tear. Detailed patient demographics can be found in Table 1. Reliability analyses indicated acceptable measurement precision for SWS and CSA outcomes in both participants with RCTs and asymptomatic participants. ICC values were good to excellent for all testing conditions and muscles ranging from 0.847–0.991. A complete breakdown of reliability values can be found in Table 2. For passive supraspinatus SWS, participants with RCTs demonstrated a baseline SEM of 0.057 m/s and an MDD of 0.541 m/s, with corresponding post-exercise values of 0.057 m/s and 0.162 m/s. In asymptomatic participants, baseline and post-exercise passive supraspinatus SWS SEM and MDD were 0.094 m/s and 0.260 m/s, respectively. For active supraspinatus SWS, baseline SEM and MDD in RCT participants were 0.090 m/s and 0.251 m/s, respectively, with post-exercise values of 0.199 m/s and 0.550 m/s. In asymptomatic participants, baseline active supraspinatus SWS SEM and MDD were 0.195 m/s and 0.541 m/s, respectively, with post-exercise values of 0.130 m/s and 0.359 m/s. Passive infraspinatus SWS measurements in participants with RCTs yielded baseline SEM and MDD values of 0.053 m/s and 0.147 m/s, respectively, with post-exercise values of 0.037 m/s and 0.104 m/s. In asymptomatic participants, baseline passive infraspinatus SWS SEM and MDD were 0.082 m/s and 0.228 m/s, respectively, and post-exercise values were 0.064 m/s and 0.177 m/s. For passive supraspinatus CSA, participants with RCTs demonstrated baseline SEM and MDD values of 0.126 cm² and 0.350 cm², respectively, with post-exercise values of 0.171 cm² and 0.475 cm². In asymptomatic participants, baseline passive supraspinatus CSA SEM and MDD were 0.110 cm² and 0.304 cm², respectively, and post-exercise values were 0.235 cm² and 0.652 cm². Active supraspinatus CSA in RCT participants yielded baseline SEM and MDD values of 0.265 cm² and 0.733 cm², respectively, with post-exercise SEM and MDD of 0.166 cm² and 0.460 cm². In asymptomatic participants, active supraspinatus CSA SEM and MDD at baseline were 0.227 cm² and 0.629 cm², respectively, with post-exercise values of 0.172 cm² and 0.476 cm².

**Table 1:** Demographic and Clinical Characteristics.

	Asymptomatic Shoulders (n = 15)	Rotator Cuff Tears (n = 17)	*p*-value
Age (years)	67.67 ± 7.67	70.47 ± 6.18	0.2684
Female (%)	44%	69%	0.3219
BMI (kg/m^2^)	26.89 ± 5.57	30.61 ± 6.60	0.0971
**Baseline Measures**			
Supraspinatus Active Mean SWS	1.49 ± 0.46 m/s	1.32 ± 0.53 m/s	0.3468
Supraspinatus Passive Mean SWS	1.43 ± 0.39 m/s	1.29 ± 0.54 m/s	0.4027
Infraspinatus Active Mean SWS	1.42 ± 0.36 m/s	1.27 ± 0.57 m/s	0.4081
Infraspinatus Passive Mean SWS	1.20 ± 0.32 m/s	1.09 ± 0.51 m/s	0.4480
Supraspinatus Active CSA	5.17 ± 0.59 cm^2^	5.13 ± 0.60 cm^2^	0.8526
Supraspinatus Passive CSA	5.56 ± 0.91 cm^2^	4.95 ± 0.85 cm^2^	0.0603
Infraspinatus Active CSA	9.45 ± 1.78 cm^2^	9.22 ± 1.92 cm^2^	0.7293
Infraspinatus Passive CSA	9.20 ± 1.65 cm^2^	8.88 ± 1.92 cm^2^	0.6117
Supraspinatus Peak Strength	76.60 ± 34.12 N	50.04 ± 20.51 N	0.0130*
Infraspinatus Peak Strength	71.57 ± 28.91 N	44.84 ± 20.77 N	0.0050*
**Post-Exercise Measures**			
Supraspinatus Passive Mean SWS	1.36 ± 0.65 m/s	0.91 ± 0.46 m/s	0.0341*
Infraspinatus Passive Mean SWS	1.19 ± 0.41 m/s	1.10 ± 0.37 m/s	0.5038
Supraspinatus Active CSA	5.75 ± 0.75 cm^2^	5.52 ± 0.48 cm^2^	0.3016
Supraspinatus Passive CSA	5.91 ± 0.85 cm^2^	4.91 ± 0.67 cm^2^	0.0009*
Infraspinatus Passive CSA	8.95 ± 1.32 cm^2^	8.50 ± 1.16 cm^2^	0.3084
Supraspinatus Peak Strength	65.03 ± 26.60 N	38.88 ± 21.66 N	0.0028*
Infraspinatus Peak Strength	64.68 ± 24.11 N	39.46 ± 18.77 N	0.0024*
Average Rating of Exertion (1–10)	1.56 ± 0.80	2.47 ± 1.56	0.0504

Values are presented as mean ± standard deviation.

Abbreviations: BMI-body mass index; SWS-shear wave speed; CSA-cross-sectional area; N-Newtons.

* Indicates statistical significance.

**Table 2:** Intraclass Correlation Coefficient (ICC), Standard Error of Measure (SEM) and Minimal Detectable Difference (MDD).

	Asymptomatic Shoulders (n = 15)			Rotator Cuff Tears (n = 17)		
	ICC_(2,3)_ (95% CIs)	SEM	MDD	ICC_(2,3)_ (95% CIs)	SEM	MDD
Supraspinatus CSA (cm^2^)	0.984 (0.963, 0.995)	0.141	0.391	0.862 (0.715, 0.947)	0.339	0.940
Supraspinatus SWS (m/s)	0.981 (0.960, 0.993)	0.094	0.260	0.985 (0.971, 0.994)	0.057	0.162
Supraspinatus CSA Active (cm^2^)	0.965 (0.925, 0.987)	0.172	0.476	0.926 (0.851, 0.970)	0.166	0.460
Supraspinatus SWS Active (m/s)	0.963 (0.913, 0.988)	0.130	0.359	0.847 (0.667, 0.946)	0.199	0.550
Infraspinatus CSA (cm^2^)	0.981 (0.961, 0.993)	0.235	0.652	0.986 (0.970, 0.995)	0.171	0.475
Infraspinatus SWS (m/s)	0.978 (0.955, 0.992)	0.064	0.177	0.991 (0.981, 0.996)	0.037	0.104

Abbreviations: ICC-Intraclass Correlation Coefficient; SEM-Standard Error of Measure; MDD-Minimum Detectable Difference; CSA-Cross-sectional Area; SWS-Shear Wave Speed.

Image testing conditions are passive unless otherwise noted.

### Shear wave speed

Mean and standard deviation of supraspinatus and infraspinatus SWS in each group and testing condition can be found in Table 1. At baseline, no significant difference was observed between groups for passive supraspinatus (*p* = 0.4027) or infraspinatus (*p* = 0.4480) shear wave speed. For supraspinatus SWS, we found a significant main effect of the exercise protocol (F = 11.29; *p* = 0.0021) on SWS with an effect size eta-squared of 0.2670. SWE muscle testing condition, i.e., active or passive, (F = 0.01; *p* = 0.9402) and rotator cuff status (F = 2.55; *p* = 0.1210) did not have a significant effect on supraspinatus SWS (Table 3). For infraspinatus SWS, we observed a significant effect of the muscle testing condition on SWS (F = 4.88; *p* = 0.0330; eta-squared = 0.1384) while the exercise protocol (F = 0.26; *p* = 0.6128) and rotator cuff status did not (F = 0.38; *p* = 0.5407). Post-hoc analysis of simple main effects revealed that, shoulders with a RCT exhibited a significantly greater reduction in passive supraspinatus shear wave speed (1.29 ± 0.54 m/s versus 0.91 ± 0.46 m/s, −34.55%; *p* = 0.0447) following the exercise protocol compared to asymptomatic shoulders, indicating increased tissue compliance (reduction in tissue stiffness) in response to acute exercise.

**Table 3:** Three-way Mixed Analysis of Variance (ANOVA) with Repeated Measures.

Effect	F	*p*-value
Dependent Variable: **Supraspinatus SWS**		
Rotator Cuff Tear	2.55	0.1210
Exercise Protocol	11.29	0.0021*
Muscle Testing Condition	0.01	0.9402
Dependent Variable: **Infraspinatus SWS**		
Rotator Cuff Tear	0.38	0.5407
Exercise Protocol	0.26	0.6128
Muscle Testing Condition	4.98	0.0330*
Dependent Variable: **Supraspinatus CSA**		
Rotator Cuff Tear	5.06	0.0320*
Exercise Protocol	12.65	0.0012*
Muscle Testing Condition	0.43	0.5157
RCT x Muscle Testing Protocol^T^	14.34	0.0007*
Dependent Variable: **Infraspinatus CSA**		
Rotator Cuff Tear	0.37	0.5469
Exercise Protocol	3.49	0.0710
Muscle Testing Condition	2.98	0.0941

SWS-shear wave speed; CSA-cross-sectional area; RCT-rotator cuff tear; F- F statistic.

^T^ Indicates a significant interaction.

* Indicates statistical significance.

### Muscle cross-sectional area

Mean and standard deviation of supraspinatus and infraspinatus CSA in each group and testing condition can be found in Table 1. For supraspinatus muscle CSA measure, we revealed significant main effects for RCT status (F = 5.06; *p* = 0.0320; eta-squared = 0.1443) and acute exercise (F = 12.65; *p* = 0.0012’ eta-squared = 0.2898), as well as a significant interaction between RCT status and SWE muscle testing condition (F = 14.34; *p* = 0.0007; eta-squared = 0.3234). No significant main effects or interactions were observed for the infraspinatus muscle CSA in relation to RCT status, pre- and post- exercise, or SWE muscle testing condition (Table 3). Post-hoc analysis revealed a significant increase in supraspinatus CSA after acute exercise in asymptomatic (5.17 ± 0.56 cm^2^ vs. 5.75 ± 0.75 cm^2^ or 11.22%; *p* = 0.0026) and symptomatic (5.13 ± 0.60 cm2 vs. 5.52 ± 0.48 cm^2^ or 7.60%; *p* = 0.0046) participants under the active condition and in asymptomatic participants under passive conditions (5.56 ± 0.91 cm^2^ vs. 5.91 ± 0.85 cm^2^ or 6.30%; *p* = 0.0020), however there was no significant difference in participants with a RCT (4.95 ± 0.85 cm^2^ vs. 4.91 ± 0.67 cm^2^ or −0.81%; *p* = 0.8900).

### Muscle strength

Baseline assessments demonstrated significant between-group differences in strength for both the supraspinatus (*p* = 0.0099) and infraspinatus (*p* = 0.0065). One participant in the RCT group was unable to generate measurable supraspinatus force against the dynamometer, however, infraspinatus force was successfully recorded. Consequently, the supraspinatus data for this participant were excluded from the final analysis, whereas the infraspinatus data were retained. Additionally, we found significant reductions in strength from pre- to post-exercise for both muscles, regardless of RCT status (Table 4). These findings indicate that both groups experienced a marked decline in peak strength of the supraspinatus and infraspinatus following the low-intensity exercise protocol.

**Table 4:** Supraspinatus and Infraspinatus Muscle Baseline and Post-Exercise Peak Strength.

	Asymptomatic Shoulders (n = 15)				Rotator Cuff Tears (n = 17)			
	Baseline	Post-Exercise	%Δ	*p-value*	Baseline	Post-Exercise	%Δ	*p-value*
Supraspinatus	76.60 ± 34.12 N	65.03 ± 26.60 N	−16.33%	0.0045*	50.04 ± 20.51 N	38.88 ± 21.66 N	−21.81%	0.0008*
Infraspinatus	71.57 ± 28.91 N	64.68 ± 24.11 N	−7.93%	0.0087*	44.84 ± 20.77 N	39.46 ± 18.77 N	−12.77%	0.0045*

Values are mean ± standard deviation.

* Statistical significance level was set at 0.05.

Abbreviations: %Δ-percent change, N-Newtons.

## Discussion

In the present study, we observed that participants with RCTs demonstrated distinct physiological responses to acute exercise compared to asymptomatic participants, characterized by a significant reduction in supraspinatus SWS (reduction in tissue stiffness) and stable supraspinatus CSA under passive testing conditions. These findings suggest that the presence of pathology may influence muscle mechanical properties, contributing to differential responses in the supraspinatus muscle during exercise, potentially indicative of maladaptive responses to exercise. Interestingly, both symptomatic and asymptomatic participants had a significant decrease in peak muscle strength following acute exercise, suggesting possible peripheral fatigue was experienced regardless of status of RCTs. The differing response in supraspinatus SWS between groups, despite the reduce peak strengths, supports the notion of maladaptive changes in the muscle’s ability to sustain mechanical properties during acute exercise with the presence of a RCT.

To our knowledge, this study is the first to examine changes in tissue stiffness and CSA of the rotator cuff muscles in response to acute exercise among shoulders with and without RCTs. Ueda et al. compared strength and CSA between symptomatic and asymptomatic patients with RCTs finding asymptomatic patients had greater muscle strength and larger CSA of the supraspinatus than symptomatic patients. [34] Zorgno et al. measured the cross-sectional area of the supraspinatus, infraspinatus, and subscapularis on MRI and found patients with adhesive capsulitis with symptoms for at least 6 months had decreased CSAs compared to matched controls. [35]. While prior studies measured CSA but not SWS, we found that supraspinatus passive mean SWS significantly decreased from baseline following the exercise protocol in participants with RCTs, while no such change was observed in those without tears. Our secondary hypothesis was partially rejected as supraspinatus CSA was significantly increased under active testing conditions regardless of rotator cuff status and under passive testing conditions in asymptomatic patients. It is not known the clinical significance. The significant decrease in supraspinatus passive mean SWS observed in participants with RCTs implies altered muscle properties that may impact force transmission capacities. Increased muscle compliance, as suggested by lower SWS, could reduce a muscle’s ability to maintain optimal tension during loading and prone to muscle fatigue or weakness. This maladaptation is further supported by the more pronounced reduction in peak strength in the RCT group compared to the asymptomatic participants. The unexpected reduction in strength within the asymptomatic group also warrants attention. Despite stable SWS values in following acute exercise, a 12% strength decline suggests that the exercise protocol, designed to mimic rehabilitative exercise intensity, induces measurable weakness even in asymptomatic population. These findings underscore the potential need for tailored recovery protocols, not only in individuals with compromised rotator cuff muscles, but also potentially in those with asymptomatic shoulders. For the RCT group, the nearly 20% decrease in peak strength is clinically relevant, emphasizing the necessity for clinicians to carefully structure progressive resistive exercise programs. Given that these participants experienced weakness even at low intensities, individualized treatment plans and specific exercise selection or prescription may be required to prevent overloading and further compromise of muscle integrity. There are potential central and peripheral factors leading to the observed changes in muscle compliance in our cohort, namely the role of pain. [36–38] While none of our participants reported pain during the exercise protocol, the average rating of perceived exertion after the exercise protocol was greater for the symptomatic cohort (2.33 ± 1.73 vs. 1.42 ± 0.96), though not statistically significant (*p* = 0.0730). An increasing body of literature indicates that chronic shoulder pain is linked to altered central nervous system processing. [39–42] The mechanisms underlying these changes, specifically, how altered pain processing and the protective response of the central nervous system to repeated painful stimuli affect muscle mechanical properties, remain largely unexplored. Data from the present study may provide a framework for future investigations into these relationships. Furthermore, changes in muscle compliance may be attributable to changes in the viscoelastic characteristics of intramuscular connective tissue in response to repeated contractions [43], however, the exact mechanisms leading to changes in muscle compliance within our population remains uncertain. Previous studies by Siracusa et al. and Andonian et al. investigated the effects of prolonged, fatigue-inducing exercise on muscle stiffness in healthy participants using SWE, and both reported significant reductions in resting muscle stiffness following exercise. [19,20] Siracusa et al. evaluated quadriceps muscle fatigue and observed a gradual decrease in resting quadriceps stiffness (*p* = 0.001), suggesting that reduced stiffness may impair force-generating capacity and contribute to peripheral fatigue. [20] Similarly, Andonian et al. assessed quadriceps muscle stiffness during and after ultramarathon running and found a significant post-race reduction compared with baseline values (*p* < 0.001). [19] The authors proposed that this decrease in stiffness was likely attributable to the supra-physiological mechanical stress associated with prolonged endurance exercise and highlighted the potential utility of SWE for monitoring pathological or maladaptive changes in muscle stiffness. SWE provides quantitative measures of the mechanical properties within the region of interest of targeted individual soft tissues. [18,44,45] Previous work has established a linear correlation between individual muscle force and dynamic shear wave modulus (µ) during contractions, indicating that shear wave modulus may serve as an indirect measure of muscle force. [46] This foundational work and work from other recent studies suggest that SWE can effectively assess changes in voluntary strength, even under fatigued or weakness conditions. [20,21,47] One factor potentially limiting the interpretation of shear modulus (µ) is the tension of the tissue of interest influences SWS; e.g., with increasing tissue tension there is an increase in SWS. [48–50] As such, care must be taken to apply minimal external pressure and ensure no to low tissue tension on the tissue(s) of interest if the results are to be expressed as shear wave modulus (µ). Conversely, in situations where the precise tissue tension cannot be measured *in-vivo*, it is advisable to present the findings in their original units of shear wave speed or speed (meters per second) to ensure a transparency with published data, as was done in the current study.

Although the scope of this study did not extend to detailed analysis of peripheral fatigue mechanisms, the pronounced strength deficits observed, despite a two-minute metabolic recovery period, suggest that muscle recovery dynamics in rotator cuff pathology may differ from normal expectations. Future research should explore the interaction between muscle compliance, SWS, and fatigue resistance to optimize rehabilitation protocols for this population. Our findings are an important step in establishing SWE as a potential tool to quantitatively measure the muscular response to exercise in patients with musculoskeletal conditions.

The current study has several strengths and limitations. Our study is the first to explore the change in rotator cuff muscle compliance in response to acute exercise in patients with and without RCTs. Our inclusion of participants with MRI-confirmation of RCTs is a methodological strength compared to clinical screening alone. One potential limitation is the mode by which we measured peak strength. Hand-held dynamometers have become increasingly popular in clinics and laboratory and while they show good validity and reliability [51,52], they lack the same rigor and standardization as isokinetic machines. [53,54] Another limitation is lack of clinical imaging in the control group to determine the status of the rotator cuff and therefore, we cannot rule out the presence of age-related asymptomatic rotator cuff pathology. Further, this study was not designed to formally measure peripheral fatigue, in part because the presence of rotator cuff pathology is known to cause pain and functional impairments, and we did not include the presence of pain for both groups to minimize the perceived pain as the confounder. During pilot testing and use of clinical experience, we discovered 20% maximal volitional contraction was sufficient to elicit patient reported fatigue but did not result in pain complaints at any point in the exercise protocol. Finally, the relatively small sample size of only twenty participants increases the potential for random variation and a higher risk of Type II errors. Future studies using comprehensive study design with randomized control trials for designing assessments of peripheral fatigue or weakness are warranted to enhance the understanding of muscle physiological conditions during various exercise protocols in patients with RCTs.

## Conclusion

Collectively, these findings suggest that supraspinatus muscle compliance remains stable in asymptomatic shoulders during acute exercise, despite transient strength loss, while increased post-exercise compliance in rotator cuff tear pathology may compromise the muscle’s capacity to sustain force production. These alterations may have implications for exercise prescription and recovery considerations in patients with rotator cuff disease. However, further research is needed to clarify the relationship among passive SWS of the rotator cuff muscles, peripheral fatigue, and the recovery timeline of exercise-induced changes in muscle compliance. Additionally, future studies should investigate the potential of SWE to quantify changes in muscle compliance following surgical intervention and during rehabilitation in this patient population.

## Supporting information

S1 File Data analyses are available through the supplemental XLSX file. (XLSX)
